# Clinical features and outcome of cryptogenic hepatocellular carcinoma compared to those of viral and alcoholic hepatocellular carcinoma

**DOI:** 10.1186/1471-2407-13-335

**Published:** 2013-07-08

**Authors:** Sang Soo Lee, Sook-Hyang Jeong, Young-Sang Byoun, Seong Min Chung, Mun Hyuk Seong, Hyung Rae Sohn, Bo-young Min, Eun Sun Jang, Jin-Wook Kim, Guan Jung Park, Yoon Jin Lee, Kyoung Ho Lee, Soyeon Ahn

**Affiliations:** 1 Department of Internal Medicine, Seoul National University Bundang Hospital, 82 Gumi-ro, 173 Beon-gil, Bundang-gu, Seongnam-si, Gyeonggi-do 463-707, South Korea; 2 Radiology, Seoul National University Bundang Hospital, Seongnam, South Korea; 3 Medical Research Collaborating Center, Seoul National University Bundang Hospital, Seongnam, South Korea

**Keywords:** Hepatocellular carcinoma, Non-alcoholic fatty liver disease, Alcohol, Visceral fat, Prognosis

## Abstract

**Background:**

Cryptogenic hepatocellular carcinoma (HCC) is thought to arise due to non-alcoholic fatty liver disease (NAFLD). This study investigated the prevalence, clinical features, and outcomes of cryptogenic HCC and compared them with those of HCC related to hepatitis B virus infection (HBV-HCC), hepatitis C virus infection (HCV-HCC), and alcohol (ALC-HCC) in Korea.

**Methods:**

The clinical features, treatment modalities, and survival data for 480 patients with HCC consecutively enrolled from January 2003 to June 2012 were analyzed. Computed tomography images were used to measure the visceral fat area (VFA) and liver-spleen density ratio.

**Results:**

Cryptogenic HCC accounted for 6.8% of all HCC cases, whereas HBV-HCC, HCV-HCC, and ALC-HCC accounted for 62.7%, 13.5%, and 10.7% of HCC cases, respectively. The cryptogenic HCC group was characterized by older age, a low proportion of male patients, a high proportion of patients with metabolic syndrome or single nodular presentation, and a low proportion of patients with portal vein invasion compared to the viral-HCC and ALC-HCC groups. However, Child Pugh classes, tumor stages, and overall survival rates of cryptogenic HCC patients were similar to those of patients with HCC of other etiologies. VFA in cryptogenic HCC patients was significantly higher than that in viral-HCC patients, but similar to that in ALC-HCC patients. The liver-spleen density ratio did not vary according to HCC etiology.

**Conclusions:**

Cryptogenic HCC accounts for approximately 7% of HCC cases in Korea, associated with an older age at diagnosis, more frequent occurrence of metabolic syndrome, and less aggressive tumor characteristics, but similar survival compared to viral-HCC or ALC-HCC. Based on VFA and the liver-to-spleen density ratio, cryptogenic HCC may be burnt-out NAFLD in which visceral fat remains but liver fat is depleted.

## Background

Hepatocellular carcinoma (HCC) is the fifth most prevalent cancer and the third most common cause of cancer mortality in the world [1]. The major underlying cause of HCC in many Asian countries is hepatitis B virus (HBV) infection, followed by hepatitis C virus (HCV) infection and alcohol [2]. However, non-alcoholic fatty liver disease (NAFLD) including non-alcoholic steato-hepatitis (NASH) increasingly appears to be a cause of HCC because of the recent global epidemic of obesity [3]. NASH can progress to cirrhosis and HCC, and a greater cause for alarm is that HCC can develop in the absence of cirrhosis when NAFLD is present [4,5]. Until now, there has been insufficient evidence to guide the surveillance examinations for early detection of HCC in the NAFLD population.

NAFLD accounts for approximately 13% of all HCC cases in the United States [6] and for 2% of cases in Japan [7]. However, once cirrhosis develops during the course of NAFLD, typical features of NASH pathology such as steatosis and inflammation often disappear, and the etiology becomes difficult to evaluate even on pathologic examination of the liver tissue. Therefore, on exclusion of HBV, HCV, alcohol, and other known etiologies such as autoimmune liver diseases or genetic liver diseases, most cases of cryptogenic cirrhosis are considered to be caused by burnt-out NASH [8]. Consequently, the clinical characteristics of cryptogenic HCC can differ from those of other HCCs that have arisen from viral or alcoholic liver disease.

In a recent study, the severity of fatty liver was positively related to visceral fat area (VFA) and the liver-to-spleen density ratio assessed by computed tomography (CT) [9], and xenon CT can be used to measure hepatic fat deposition in NASH patients [10]. However, because VFA and the liver-to-spleen ratio have not been studied in HCC patients until now, we undertook a comparative analysis of VFA and the liver-to-spleen density ratio according to different HCC etiologies.

The aim of present study was to investigate the prevalence, characteristics, and outcomes of cryptogenic HCC and to compare them with those of HBV-associated HCC (HBV-HCC), HCV-associated HCC (HCV-HCC), and alcohol-associated HCC (ALC-HCC).

## Methods

### Subjects and etiologic classification of HCC

The subjects were 512 patients consecutively diagnosed with HCC in Seoul National University Bundang Hospital from January 2003 to June 2012. The diagnosis of HCC was based on histology or typical radiographic findings, which are hepatic nodules with arterial enhancement and venous wash-out on contrast-enhanced CT or magnetic resonance imaging (MRI) [11,12].

HCC was classified etiologically as HBV-HCC (hepatitis B surface antigen, HBsAg positivity), HCV-HCC (anti-HCV positivity), ALC-HCC (intake of more than 40 g/day of alcohol for men and 20 g/day for women for more than 10 years) [3], and cryptogenic HCC. Cryptogenic HCC diagnosis was an exclusion diagnosis according to the criteria for NAFLD [6], which was based on the following: (1) absence of serologic or clinical evidence of HBV or HCV infection; (2) alcohol consumption < 20 g/day in men (< 10 g/day in women); and (3) no evidence of other causes of chronic liver disease such as autoimmune hepatitis, drug-induced hepatitis, hemochromatosis, Wilson’s disease, intestinal bypass surgery, Budd-Chiari syndrome, primary biliary cirrhosis, and primary sclerosing cholangitis. We excluded 32 patients with combined etiologies, Budd-Chiari syndrome, or autoimmune hepatitis and the remaining 480 patients were finally enrolled in this study.

### Comparative analyses of clinical variables, treatment modalities, and survival data of cryptogenic HCC

Retrospective analyses of demographic data, comorbid conditions, clinical and pathological data, tumor characteristics on radiologic images, treatment modalities, and survival rates were performed, comparing cryptogenic HCC with HBV-HCC, HCV-HCC, or ALC-HCC. This study was approved by the Institutional Review Board of the Seoul National University Bundang Hospital.

Clinical data on height; weight; body mass index (BMI); hypertension; diabetes; hyperlipidemia; HBsAg positivity; anti-HBsAg positivity; anti-HCV positivity; levels of triglyceride (TG), total cholesterol, low-density lipoprotein cholesterol (LDL), high-density lipoprotein cholesterol (HDL), total protein, serum albumin, alanine aminotransferase (ALT), aspartate aminotransferase (AST), γ glutamyl transferase, and alkaline phosphatase (ALP); platelet count, hemoglobin levels, serum sodium levels, creatinine levels, and alpha-fetoprotein (AFP) levels were retrieved from electronic records, and the Child-Pugh class and model for end-stage liver disease (MELD) score were calculated. Obesity was defined as a BMI of ≥25 kg/m^2^ according to the criterion of the World Health Organization and the National Institute of Health for obesity in Asian populations [13].

To analyze tumor characteristics, the number and diameter of HCC nodules, the presence of vascular invasion, tumor stage represented as Barcelona Clinic Liver Cancer (BCLC) and TNM stages [14,15], and presence of accompanying cirrhosis in the non-tumorous liver were evaluated. Liver cirrhosis was defined by the presence of portal hypertension manifested as varices, splenomegaly, ascites, or hepatic encephalopathy and compatible imaging findings accompanied by throm-bocytopenia (<100,000/μl) [11].

Treatment modalities for HCC were classified as surgical resection, transarterial chemoembolization (TACE), radiofrequency ablation (RFA), percutaneous ethanol injection (PEI), systemic chemotherapy, targeted agent (sorafenib), and liver transplantation during the follow-up period. Overall survival of the cryptogenic HCC patients was compared with that of viral or alcoholic HCC patients.

### Measurement of visceral fat area and the liver-to-spleen density ratio on CT images

To investigate the association between NAFLD and cryptogenic HCC, VFA and the liver-to-spleen density ratio were measured in 113 patients by analyzing CT images obtained at the level of the umbilicus using commercially available software (Rapidia 2.8; INFINITT, Seoul, Korea). VFA was defined as the sum of intraperitoneal fat area with attenuation between -250 and -50 Hounsfield units (HU) on a pre-enhanced liver CT scan. The liver attenuations were measured from 4 regions of interest (ROIs) of 100 mm^2^ in segments 5, 6, 7, and 8 of the liver and spleen attenuations were measured as the mean HU of 2 ROI values in the spleen. Using these values, we determined the liver-to-spleen density ratio. ROIs in the liver and spleen were selected in peripheral areas away from major vessels and tumors. VFA and the liver-to-spleen density ratio were compared between 18 cryptogenic HCC patients and 36 randomly selected age- and sex-matched HBV-HCC patients or 36 randomly selected age- and sex-matched HCV-HCC patients. However, because the 23 ALC-HCC patients with available VFA data were mostly male, sex matching was not possible. Further, accurate age- and sex-matching was not possible because of the small sample size of each subgroup of HCC patients.

### Statistical analysis

For comparative analysis, the Mann-Whitney test was used for quantitative variables and the chi-square test or 2-tailed Fischer’s exact test was used for qualitative variables. Overall survival and disease free survival according to HCC etiology were compared using the Kaplan-Meier model and log-rank test. A significant p value was defined as < 0.05 and all statistical analyses were performed using PASW software (Version 18, SPSS Inc, Chicago, IL, USA).

## Results

### The prevalence of cryptogenic HCC in Korea

**Figure 1:**
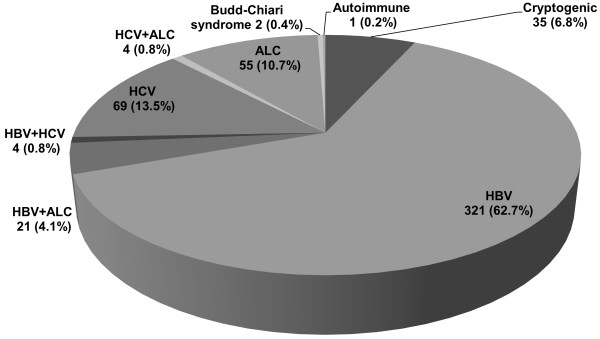
**The prevalence of cryptogenic hepatocellular carcinoma (HCC).** The major underlying cause of HCC in Korea is hepatitis B virus (HBV) infection followed by hepatitis C virus (HCV) infection and alcohol (ALC). Cryptogenic HCC accounts for 6.8% of HCC cases.

**Figure 2:**
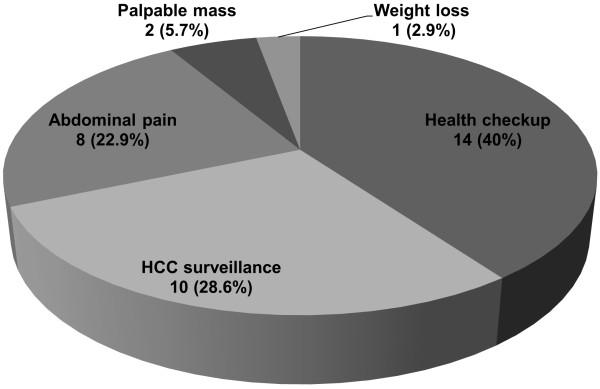
**Initial presentation at diagnosis of hepatocellular carcinoma (HCC) in 35 patients with cryptogenic HCC.** Of these, only 11 patients presented with symptoms such as abdominal pain, palpable mass, and weight loss.

Of the 512 HCC cases, HBV-HCC accounted for 321 (62.7%), HCV-HCC accounted for 69 (13.5%), ALC-HCC accounted for 55 (10.7%), and cryptogenic HCC accounted for 35 (6.8%) (Figure 1). Of 35 patients with cryptogenic HCC, 14 were asymptomatic and were diagnosed during a health check-up, 10 patients with liver cirrhosis were diagnosed during a surveillance examination, and the remaining 11 patients (31.4%) presented with symptoms such as abdominal pain (n = 8), a palpable mass (n = 2) or weight loss (n = 1) (Figure 2).

### Clinical features of cryptogenic HCC compared to HCC caused by other etiologies

The clinical characteristics of HCC according to etiology are summarized in Table 1. Cryptogenic HCC was diagnosed at a significantly older age (68.3 ± 10.5 years) than HBV-HCC (57.1 ± 10.3 years, p < 0.001) and a significantly lower proportion of patients were male (57.1%) compared to ALC-HCC (92.7%, p < 0.001) or HBV-HCC (76.0%, p = 0.015) patients. Twenty-six patients (74.3%) with cryptogenic HCC had one or more components of metabolic syndrome, showing a higher prevalence of obesity (45.7%), diabetes (37.1%), hypertension (57.1%), and hyperlipidemia (25.7%) compared to patients with other etiologies. Although serum hemoglobin levels were significantly lower in cryptogenic HCC patients than in HBV-HCC patients and serum creatinine levels were significantly lower in cryptogenic HCC patients than HCV-HCC patients, whereas other laboratory tests and liver function as assessed by Child-Pugh class and the MELD score did not differ significantly according to HCC etiology.

**Table 1:** Comparison of demographic data, comorbidities, and laboratory data between cryptogenic hepatocellular carcinoma (HCC) patients and patients with HCC associated with hepatitis B virus infection, hepatitis C virus infection, and alcohol

	**Cryptogenic HCC (n = 35)**	**HBV-HCC**	* p	**HCV-HCC**	**p	**ALC-HCC**	***p
		**(n = 321)**		**(n = 69)**		**(n = 55)**	
Age, yr†	68.3 ± 10.5	57.1 ± 10.3	**<0.001**	68.1 ± 9.0	0.896	65.5 ± 10.0	0.164
Gender, Male††	20 (57.1)	244 (76.0)	**0.015**	44 (63.8)	0.512	51 (92.7)	**<0.001**
BMI (kg/m^2^) †	24.6 ± 3.3	23.4 ± 3.4	**0.034**	22.2 ± 3.9	**0.002**	24.0 ± 4.3	0.143
Obesity††	16 (45.7)	85 (26.7)	**0.018**	13 (18.8)	**0.004**	12 (21.8)	**0.017**
Diabetes ††	13 (37.1)	55 (17.1)	**0.004**	17 (24.6)	0.183	19 (34.5)	0.802
Hypertension ††	20 (57.1)	77 (24.0)	**<0.001**	32 (46.4)	0.299	18 (32.7)	**0.022**
Hyperlipidemia††	9 (25.7)	10 (3.1)	**<0.001**	4 (5.8)	**0.004**	2 (3.6)	**0.003**
Follow-up period, mo†	26.1 ± 27.8	28.5 ± 27.0	0.573	29.9 ± 25.1	0.362	29.2 ± 25.6	0.518
Albumin (g/dL) †	3.7 ± 0.5	3.8 ± 0.6	0.346	3.7 ± 0.6	0.780	3.7 ± 0.5	0.545
Bilirubin (mg/dL) †	1.2 ± 0.7	1.6 ± 2.4	0.437	1.8 ± 2.7	0.551	1.6 ± 1.9	0.216
ALP (IU/L) †	123.3 ± 73.3	137.2 ± 97.2	0.555	125.2 ± 63.1	0.728	132.6 ± 91.8	0.855
AST (IU/L) †	83.8 ± 165.4	89.3 ± 140.5	0.342	111.9 ± 114.6	**<0.001**	53.2 ± 31.9	0.419
ALT (IU/L) †	58.7 ± 99.0	64.4 ± 98.4	0.111	78.6 ± 60.6	**0.001**	47.4 ± 72.7	0.371
Platelet (10^3^/μL) †† (<10/10 ~ 13/>13)	5/7/23	87/50/182	0.157	19/18/32	0.056	14/9/42	0.263
PT-INR†	1.14 ± 0.16	1.15 ± 0.18	0.897	1.15 ± 0.14	0.791	1.14 ± 0.19	0.593
Hemoglobin (g/dL) †	12.6 ± 2.3	13.6 ± 2.0	**0.007**	12.8 ± 2.1	0.570	13.1 ± 2.2	0.460
Na (mmol/L) †	137.0 ± 8.8	137.4 ± 9.6	0.551	137.7 ± 4.0	0.535	137.9 ± 3.4	0.340
Creatinine (mg/dL) †	0.9 ± 0.2	1.0 ± 0.3	0.738	1.2 ± 1.0	**0.040**	1.2 ± 1.0	0.537
C-P class (A/BC) ††	29/6	246/75	0.404	50/19	0.241	40/15	0.268
MELD score†	9.0 ± 2.9	9.6 ± 3.2	0.273	10.2 ± 3.7	0.076	10.5 ± 4.3	0.051

†Expressed as the mean ± standard deviation. ††Expressed as the number of subjects (%).

Values in bold, statistically significant (p < 0.05).

*BMI*, Body mass index; *AST*, Aspartate transaminase; *ALT*, Alanine transaminase; *PT-INR*, Prothrombin time-international normalized ratio; *C-P class*, Child-Pugh class; *MELD*, Model for end-stage liver disease; *HBV-HCC*, Hepatitis B-associated hepatocellular carcinoma; *HCV-HCC*, Hepatitis C-associated hepatocellular carcinoma; *ALC-HCC*, Alcohol-associated hepatocellular carcinoma.

*p: Cryptogenic HCC vs. HBV-HCC.

**p: Cryptogenic HCC vs. HCV-HCC.

***p: Cryptogenic HCC vs. ALC-HCC.

Tumor characteristics and stage for cryptogenic HCC are summarized in Table 2. Cryptogenic HCC presented as a solitary nodule more frequently (74.3%) than HBV-HCC (53.3%, p = 0.026), HCV-HCC (49.3%, p = 0.020), and ALC-HCC (36.4%, p = 0.002). Moreover, it presented with a significantly lower rate of portal vein thrombosis (11.4%) than HBV-HCC (27.7%, p = 0.037) and HCV-HCC (31.9%, p = 0.023). However, no significant differences were noted in tumor stage in terms of BCLC and TNM stages between cryptogenic HCC and other HCCs.

**Table 2:** Comparison of tumor characteristics and treatment modalities between cryptogenic hepatocellular carcinoma (HCC) patients and patients with HCC associated with hepatitis B virus infection, hepatitis C virus infection, and alcohol

	**Cryptogenic HCC (n = 35)**	**HBV-HCC**	* p	**HCV-HCC**	**p	**ALC-HCC**	***p
		**(n = 321)**		**(n = 69)**		**(n = 55)**	
AFP (IU/mL)			**0.036**		0.938		0.116
<20	19 (57.6)	108 (34.4)		33 (53.2)		35 (70.0)	
20 ~ 200	4 (12.1)	78 (24.8)		12 (19.4)		8 (16.0)	
>200	10 (30.3)	128 (40.8)		17 (27.4)		7 (14.0)	
PVT	4 (11.4)	89 (27.7)	**0.037**	22 (31.9)	**0.023**	10 (18.2)	**0.389**
Extrahepatic metastasis	1 (2.9)	19 (5.9)	0.706	5 (7.2)	0.661	3 (5.5)	0.560
TNM stage			0.361		0.889		0.385
I	16 (45.7)	121 (37.7)		34 (49.3)		15 (27.3)	
II	5 (14.3)	57 (17.8)		10 (14.5)		20 (36.4)	
III	13 (37.1)	122 (38.0)		20 (29.0)		17 (30.9)	
IV	1 (2.9)	21 (6.5)		5 (7.2)		3 (5.5)	
BCLC stage			0.120		0.380		0.120
A	18 (51.4)	148 (46.1)		38 (55.1)		23 (41.8)	
B	11 (31.4)	65 (20.2)		8 (11.6)		15 (27.3)	
C	6 (17.1)	94 (29.3)		20 (29.0)		13 (23.6)	
D	0 (0)	14 (4.4)		3 (4.3)		4 (7.3)	
HCC nodules			**0.026**		**0.020**		**0.002**
1	26 (74.3)	171 (53.3)		34 (49.3)		20 (36.4)	
2 ~ 3	4 (11.4)	63 (19.6)		14 (20.3)		17 (30.9)	
≥4	5 (14.3)	87 (27.1)		21 (30.4)		18 (32.7)	
Largest tumor size			0.536		0.517		0.940
<2cm	10 (28.6)	73 (22.7)		27 (39.1)		15 (27.3)	
2 ~ 5cm	12 (34.3)	120 (37.4)		17 (24.6)		21 (38.2)	
>5cm	13 (37.1)	128 (39.9)		25 (36.2)		19 (34.5)	
Treatment modality							
Resection	4 (11.4)	45 (14.0)	0.801	7 (10.1)	0.841	8 (14.5)	0.672
RFA	9 (25.7)	111 (34.6)	0.349	28 (40.6)	0.135	15 (27.3)	0.871
TACE	28 (80.0)	262 (81.6)	0.820	54 (78.3)	0.837	46 (83.6)	0.660
PEI	1 (2.9)	10 (3.1)	1.000	2 (2.9)	1.000	1 (1.8)	1.000
Systemic chemotherapy	2 (5.7)	24 (7.5)	1.000	1 (1.4)	0.261	3 (5.3)	1.000
Sorafenib	3 (8.6)	25 (7.8)	0.747	7 (10.1)	1.000	6 (10.9)	0.719
Liver transplantation	0 (0.0)	6 (1.9%)	1.000	0 (0.0)		1 (1.8%)	1.000
No treatment	2 (5.7)	35 (10.4)	0.558	7 (10.1)	0.714	7 (12.7)	0.473

Data are expressed as the number (%). Values in bold, statistically significant (p < 0.05).

*AFP*, Alpha-fetoprotein; *PVT*, Portal vein thrombosis; *BCLC*, Barcelona Clinic Liver Cancer; *HCC*, Hepatocellular carcinoma; *RFA*, Radiofrequency ablation; *TACE*, Transarterial chemoembolization; *PEI*, Percutaneous ethanol injection; *HBV-HCC*, Hepatitis B-associated hepatocellular carcinoma; *HCV-HCC*, Hepatitis C-associated hepatocellular carcinoma; *ALC-HCC*, Alcohol-associated hepatocellular carcinoma.

*p: Cryptogenic HCC vs. HBV-HCC.

**p: Cryptogenic HCC vs. HCV-HCC.

***p: Cryptogenic HCC vs. ALC-HCC.

Of 35 patients with cryptogenic HCC, 27 (77.1%) had liver cirrhosis according to clinical or image findings. No significant differences were noted in age, sex, BMI, tumor size, and number of nodules between 8 non-cirrhotic HCC patients and 27 cirrhotic patients (Table 3).

**Table 3:** Demographics and tumor characteristics for cryptogenic hepatocellular carcinoma patients stratified by the presence of liver cirrhosis

	**No cirrhosis (n = 8)**	**Cirrhosis (n = 27)**	**p**
Age (yr) †	60.8 ± 16.8	70.5 ± 6.7	0.080
Gender, male††	7 (87.5)	13 (48.1)	0.101
BMI (kg/m^2^) †	23.0 ± 1.9	25.1 ± 3.5	0.104
HCC nodules			0.080
1	8	18	
2 ~ 3	0	4	
≥4	0	5	
Largest tumor size			0.518
<2cm	3	7	
2 ~ 5cm	0	12	
>5cm	5	8	
PVT††	0 (0)	4 (14.8)	0.553

†Expressed as the mean ± standard deviation. ††Expressed as the number of subjects (%).

*BMI*, Body mass index; *HCC*, Hepatocellular carcinoma; *PVT*, Portal vein thrombosis.

### Treatment modalities and survival outcomes

**Figure 3:**
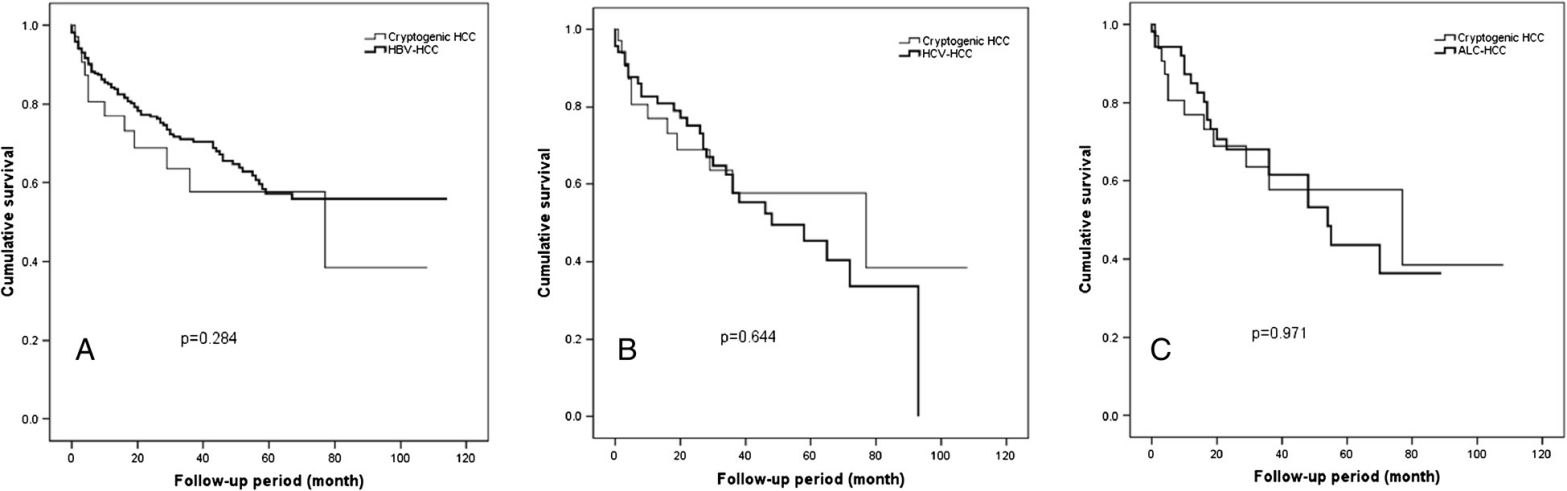
**Overall survival time of hepatocellular carcinoma (HCC) patients according to etiology.** Comparisons of the cumulative survival of the 35 cryptogenic HCC patients with that of 321 hepatitis B virus-associated HCC (HBV-HCC) patients **(A)**, 69 hepatitis C virus-associated HCC (HCV-HCC) patients **(B)**, and 55 alcohol-associated HCC (ALC-HCC) patients **(C)** are shown.

**Figure 4:**
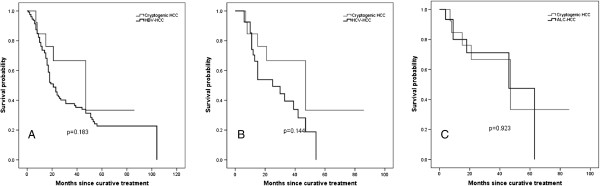
**Disease free survival of hepatocellular carcinoma (HCC) patients according to etiology.** There were no significant differences in disease free survival between cryptogenic HCC patients (n = 15) and patients with HCC associated with hepatitis B virus infection **(A**, n = 128**)**, hepatitis C virus infection **(B**, n=29**)**, and alcohol **(C**, n=15**)**.

No differences were noted in treatment modalities between cryptogenic HCC and other HCC patients (Table 2). Comparative analyses of overall survival showed no significant differences between cryptogenic HCC and HCC patients with other etiologies (Figure 3). Causes of death in cryptogenic HCC patients were hepatic failure (n = 8) and HCC progression (n = 4), and none of the cryptogenic HCC patients died due to a cardiovascular event. In addition, disease free survival defined as the duration from date of curative-intent treatment to date of local and/or distant recurrence or death of 187 patients who underwent curative treatment was obtained, which showed no significant differences between cryptogenic HCC patients (n = 15) and HBV-HCC (n = 128), HCV-HCC (n = 29), and ALC-HCC (n = 15) (Figure 4).

### Measurement of VFA and the liver-to-spleen density ratio

VFA and the liver-to-spleen density ratio were measured in 18 patients with cryptogenic HCC who were age- and sex-matched with 36 HBV-HCC patients and 36 HCV-HCC patients (Table 4). The cryptogenic HCC patients showed significantly higher VFA (134.4 ± 53.9 cm^2^) than the HBV-HCC (100.0 ± 51.3 cm^2^, p = 0.037) and HCV-HCC (105.0 ± 51.1 cm^2^, p = 0.034) patients, whereas VFA did not differ between cryptogenic HCC and ALC-HCC patients. In contrast, the liver-to-spleen density ratio did not differ between the cryptogenic HCC patients and the HBV-HCC, HCV-HCC, and ALC-HCC patients.

**Table 4:** Comparison of visceral fat area and the liver-to-spleen density ratio between cryptogenic hepatocellular carcinoma (HCC) patients and patients with HCC of other etiologies

	**§Cryptogenic HCC (n = 18)**	**HBV-HCC (n = 36)**	*p	**HCV-HCC (n = 36)**	**p	**¶ ALC-HCC**	***p
						**(n = 23)**	
Age (yr) †	70.3 ± 10.6	67.4 ± 9.1	0.201	69.0 ± 8.8	0.646	67.7 ± 9.6	0.510
Gender, male††	9 (50.0)	9 (50.0)	1.000	9 (50.0)	1.000	18 (78.3)	0.097
BMI (kg/m^2^) †	24.7 ± 3.0	23.3 ± 3.1	0.196	22.8 ± 3.2	0.050	24.5 ± 4.9	0.415
VFA (cm^2^) †	134.4 ± 53.9	100.0 ± 51.3	**0.037**	105.0 ± 51.1	0.034	136.9 ± 64.9	0.599
Liver attenuation (HU) †	54.3 ± 5.5	53.7 ± 5.8	0.533	53.5 ± 6.7	0.441	50.7 ± 8.3	0172
Spleen attenuation (HU) †	44.0 ± 4.5	43.7 ± 4.0	0.263	45.0 ± 3.8	0.734	44.0 ± 5.0	0.743
Liver/Spleen ratio†	1.24 ± 0.14	1.23 ± 0.14	0.854	1.20 ± 0.14	0.419	1.15 ± 0.14	0.066

†Expressed as the mean ± standard deviation. ††Expressed as the number of subjects (%). Values in bold, statistically significant (p < 0.05).

§ Available data in 18 patients. Eighteen patients with cryptogenic HCC were matched for age and sex (1:2 ratio) with HBV-HCC or HCV-HCC patients.

¶ Of 55 ALC-HCC, VFA was measured in 23 patients. ALC-HCC patients did not match with regard to age and sex with cryptogenic-HCC patients because they were mostly of male gender and because the sample sizes of each subgroup were small.

*HU*, Hounsfield unit; *VFA*, Visceral fat accumulation; *BMI*, Body mass index.

*p: Cryptogenic HCC vs. HBV-HCC.

**p: Cryptogenic HCC vs. HCV-HCC.

***p: Cryptogenic HCC vs. ALC-HCC.

## Discussion

Cryptogenic HCC accounted for 6.8% of HCC cases in this study. Compared to HCC of other etiologies, the clinical features and outcomes associated with cryptogenic HCC were older age, accompanying metabolic syndrome, single nodules, and less portal vein invasion, although Child Pugh classes, tumor stages, and overall cumulative survival rates did not differ significantly. Interestingly, VFA and the liver-to-spleen density ratio measurements supported the theory that cryptogenic HCC may be burnt-out NAFLD with residual visceral fat but with depleted liver fat.

The prevalence of NAFLD is approximately 18% in Korea [16,17], with a rapidly increasing tendency as in Western countries. NAFLD is considered an important etiology of HCC since Powell et al. reported the first case of NAFLD-associated HCC (NAFLD-HCC) [18,19], although a recent systematic review showed that the risk for HCC was substantially lower for NASH cirrhosis than for HCV-related cirrhosis [20]. This study showed that cryptogenic HCC accounted for 6.8% of current HCC cases in Korea, a prevalence lower than that in the United States (13%) [6], but similar to that in Japan (7.1%) and Italy (7%) [7,21]. However, the true prevalence of NAFLD-HCC is difficult to evaluate because liver fat storage is depleted as hepatic fibrosis progresses, a process known as burnt-out NASH, which makes diagnosis difficult.

NAFLD is the hepatic manifestation of metabolic syndrome accompanied by high rates of obesity, diabetes, hyperlipidemia, and hypertension. As expected, patients with cryptogenic HCC showed a higher prevalence of metabolic syndrome than those with other HCCs, with the highest mean age at diagnosis and lowest proportion of male subjects compared to all other HCC groups. In previous studies, patients with cryptogenic cirrhosis were approximately 3 years older than patients with HCV at the time of HCC diagnosis [4,22], and the proportion of male patients with NASH-associated cirrhosis was lower than that of female patients. However, NAFLD-HCC showed a modest male predominance, suggesting a higher carcinogenic potential in male patients [23,24].

Approximately 30% of patients did not realize that they had HCC until symptomatic presentation, whereas 30% of cryptogenic HCC patients had their tumors detected during surveillance. In a previous study, HCC patients who were diagnosed through surveillance had smaller tumors at diagnosis and were likely to be eligible for surgical resection or RFA therapies, resulting in survival improvement [6]. In our study, 9 patients with cryptogenic HCC who were diagnosed during surveillance had tumors less than 5 cm in diameter, with AFP levels lower than 20 IU/mL (data not shown). Although the cryptogenic HCC patients showed similarly elevated aminotransferase levels at initial diagnosis, NASH patients may commonly have severe liver disease even when aminotransferase levels are within the normal range [25]. Therefore, further studies are needed to determine surveillance indicators for cryptogenic HCC.

Approximately 25% of cryptogenic HCC patients in this study did not show clinical or histological evidence of cirrhosis, findings comparable with those of a cross-sectional study in Japan where 43 of 87 patients histologically proven to have NASH-associated HCC had no established cirrhosis [24]. Since 2004, at least 116 cases of biopsy-confirmed NAFLD-associated HCC without cirrhosis have been reported, suggesting that non-cirrhotic HCC may be a more common feature in NAFLD-associated HCC than in viral- or ALC-HCC [26].

The clinical characteristics and disease course of cryptogenic HCC was found to be more indolent than HCC caused by viral or alcoholic liver disease. According to our results, cryptogenic HCC presented as a solitary nodule more frequently and with a significantly lower rate of portal vein thrombosis than HBV-HCC, HCV-HCC, and ALC-HCC. This is similar to the results of previous studies, which documented that HCC nodules in patients with NAFLD tended to be solitary lesions [27,28] or single, large, or well-differentiated tumors [23,27,28], suggesting that NAFLD-HCC has less severe tumor characteristics than HCC caused by virus infection or alcohol. Single nodular presentation may be related to a lower tendency of portal vein invasion and, subsequently, less intrahepatic metastasis. Additionally, no significant difference was noted in Child Pugh classes, tumor stages, and overall survival rates between the cryptogenic HCC patients and the other HCC patients, a finding partially concordant with other reports [29-31]. In recent studies, patients with cryptogenic HCC had a higher survival rate than those with HCV-HCC and/or ALC-HCC after curative treatment [29,30]. This discrepancy may be related to the patients’ characteristics of cryptogenic HCC, such as older age and more frequent occurrence of metabolic syndrome and other comorbidity, in spite of the less aggressive behavior of tumors in cryptogenic HCC.

The most remarkable finding of the present study was that VFA in cryptogenic HCC patients was higher than that in either HBV-HCC or HCV-HCC patients, whereas no significant difference was observed between cryptogenic HCC and ALC-HCC patients. In contrast, no difference was noted in the liver-to-spleen density ratio according to HCC etiology. As previously reported, the severity of fatty liver is positively correlated with VFA, whereas VFA and the liver-spleen density ratio are negatively correlated [9]. Moreover, the liver-to-spleen density ratio tends to be lower in cirrhosis patients than in non-cirrhotic NASH patients [10]. As hepatic fibrosis progresses, the liver-to spleen density ratio may decrease because liver fat storage is depleted, but visceral fat is preserved. Therefore, our results suggest that cryptogenic HCC may be associated with burnt-out NAFLD.

This study had several limitations because it was based on a retrospective single-center design with a small sample size. NAFLD was histologically proven only in 7 patients out of 35 with cryptogenic HCC. Body mass index or alanine aminotransferase levels may fluctuate in these patients during their lifetime, but retrospective nature of this study could not show those clinical features. Furthermore, in the context of occult HBV infection in hepatocarcinogenesis, anti-HBc and HBV DNA were not completely examined in all of the cryptogenic HCC patients. In this study, 9 out of 35 cryptogenic HCC patients showed anti-HBc positivity, but most of them showed serum HBV DNA negativity, which may suggest a trivial role for occult HBV infection in cryptogenic HCC.

## Conclusions

Cryptogenic HCC accounts for approximately 7% of HCC cases in Korea and is associated with an older age at diagnosis, high prevalence of metabolic syndrome, and less aggressive tumor characteristics compared to viral-HCC and ALC-HCC, but a similar overall survival rate. VFA and the liver-spleen density ratio measurements support the theory that most cryptogenic HCC cases may be associated with burnt-out NAFLD. Surveillance for the early detection of HCC in NAFLD patients should be considered.

## Abbreviations

HCC: Hepatocellular carcinoma; HBV: Hepatitis B virus; HCV: Hepatitis C virus; NAFLD: Non-alcoholic fatty liver disease; NASH: Non-alcoholic steatohepatitis; VFA: Visceral fat area; CT: computed tomography; HBV-HCC: Hepatitis B virus -associated hepatocellular carcinoma; HCV-HCC: Hepatitis C virus -associated hepatocellular carcinoma; ALC-HCC: Alcohol-associated hepatocellular carcinoma; MRI: Magnetic resonance imaging; BMI: Body mass index; TG: Triglyceride; LDL: Low-density lipoprotein; HDL: High-density lipoprotein cholesterol; ALT: Alanine aminotransferase; AST: Aspartate aminotransferase; ALP: Alkaline phosphatase; AFP: Alpha-fetoprotein; MELD: Model for end-stage liver disease; BCLC: Barcelona Clinic Liver Cancer; TACE: Transarterial chemoembolization; RFA: Radiofrequency ablation; PEI: Percutaneous ethanol injection; HU: Hounsfield units; ROI: Regions of interest; NAFLD-HCC: Non-alcoholic fatty liver disease -associated hepatocellular carcinoma.

## Competing interest

The authors declare that they have no competing interests.

## Authors’ contributions

SSL: study design and concept, data acquisition, and drafting of the manuscript, YSB, SMC, MHS, HRS, and BYM: study concept and design and data acquisition, ESJ and JWK: study supervision and critical review of the manuscript, GJP, YJL, KHL and SA: study concept and design and data acquisition, SHJ: study concept and design, data acquisition and interpretation, study supervision, and critical review of the manuscript. All authors read and approved the final manuscript.

## Pre-publication history

The pre-publication history for this paper can be accessed here:

http://www.biomedcentral.com/1471-2407/13/335/prepub
